# Mutations in the Motile Cilia Gene *DNAAF1* Are Associated with Neural Tube Defects in Humans

**DOI:** 10.1534/g3.116.033696

**Published:** 2016-08-18

**Authors:** Chunyue Miao, Qian Jiang, Huili Li, Qin Zhang, Baoling Bai, Yihua Bao, Ting Zhang

**Affiliations:** * Beijing Municipal Key Laboratory of Child Development and Nutriomics, Capital Institute of Pediatrics-Peking University Teaching Hospital, 100020, China; † Department of Medical Genetics, Beijing Municipal Key Laboratory of Child Development and Nutriomics, Capital Institute of Pediatrics, 100020, China; ‡ Beijing Municipal Key Laboratory of Child Development and Nutriomics, Capital Institute of Pediatrics, 100020, China

**Keywords:** neural tube defects, motile cilia, *DNAAF1*, loss-of function

## Abstract

Neural tube defects (NTDs) are severe malformations of the central nervous system caused by complex genetic and environmental factors. Among genes involved in NTD, cilia-related genes have been well defined and found to be essential for the completion of neural tube closure (NTC). We have carried out next-generation sequencing on target genes in 373 NTDs and 222 healthy controls, and discovered eight disease-specific rare mutations in cilia-related gene *DNAAF1*. DNAAF1 plays a central role in cytoplasmic preassembly of distinct dynein-arm complexes, and is expressed in some key tissues involved in neural system development, such as neural tube, floor plate, embryonic node, and brain ependyma epithelial cells in zebrafish and mouse. Therefore, we evaluated the expression and functions of mutations in *DNAAF1* in transfected cells to analyze the potential correlation of these mutants to NTDs in humans. One rare frameshift mutation (p.Gln341Argfs*10) resulted in significantly diminished DNAAF1 protein expression, compared to the wild type. Another mutation, p.Lys231Gln, disrupted cytoplasmic preassembly of the dynein-arm complexes in cellular assay. Furthermore, results from NanoString assay on mRNA from NTD samples indicated that DNAAF1 mutants altered the expression level of NTC-related genes. Altogether, these findings suggest that the rare mutations in *DNAAF1* may contribute to the susceptibility for NTDs in humans.

Neural tube defects (NTDs) occur during the embryogenic period when the neural tube fails to close normally, and affects about 0.5–2 of every 1000 pregnancies worldwide each year (Mitchell 2005; Liu *et al.* 2016; Seidahmed *et al.* 2014). NTDs are a series of anomalies in the development of the central nervous system (Greene and Copp 2014), during which the formation of neural tubes is a continuous and intricate process. Initially, the dorsal neuroepithelium thickens to form the neural plate, then bends to form neural folds, and finally fuses and completely closes in the dorsal midline (Greene and Copp 2009). Failure of the complex procedure of neural tube closure (NTC) resulted in a wide variation of clinical severity among the NTDs, including anencephaly, myelomeningocele, spina bifida, and myeloschisis (Copp and Greene 2013).

Both genetic and environmental factors are involved in the etiology of NTDs. Genetic factors may contribute up to 70% of NTD prevalence, based on epidemiological evidences to date (Leck 1974; Copp and Greene 2010), and have been classified into several functional categories. Among them, cilia-related genes have been recognized as one of the categories essential to the success of NTC (May-Simera and Kelley 2012; Bay and Caspary 2012; Murdoch and Copp 2010; Harris and Juriloff 2010; Copp and Greene 2013; Wallingford *et al.* 2013). Cilia are microtubule-based filamentous organelles that are conserved evolutionarily in eukaryotes and play vital roles in diverse biological processes during embryogenesis and the maintenance of organ integrity (Fliegauf *et al.* 2007). Disruption of cilia structure in N-ethyl-N-nitrosourea–induced adenosine diphosphate ribosylation factor-like 13B (*Arl13b*) mouse mutants leads to exencephaly and spina bifida, phenotypes of NTDs (Caspary *et al.* 2007). In a previous study, 30% (28 of 93) of homozygous mouse gene-trap C2 calcium-dependent domain containing 3 (*C2cd3*; required for ciliogenesis in the mouse) mutants exhibited exencephaly in the midbrain and posterior forebrain (Hoover *et al.* 2008). Other studies have shown that a proportion of homozygous mouse embryos for *Ift172* (Huangfu *et al.* 2003; Huangfu and Anderson 2005) and *Dync2h1* (Huangfu and Anderson 2005) mutants exhibit exencephaly stemming from disrupted ciliogenesis or cilia function. A study on motile cilia-related gene *Foxj1*, a forkhead family transcription factor (Yu *et al.* 2008), suggested that the architecture and morphology of neural tube motile cilia could affect the transduction of sonic hedgehog (SHH) signaling (Cruz *et al.* 2010). To date, approximately 20 cilia-related genes have been reported to be strongly associated with NTC in mice (Murdoch and Copp 2010; Harris and Juriloff 2010; Wallingford *et al.* 2013; May-Simera and Kelley 2012; Louvi and Grove 2011). Results from our previous study suggest that ciliogenic copy number variants are associated with NTDs in humans (Chen *et al.* 2013), indicating that variants in cilia-related genes may lead to NTDs in humans. This is supported by a recent clinical report that a human fetus with mutations in *CEP290*, an important component of the cilium, manifested occipital meningoencephalocele, hydrocephalus, and other defects (Molin *et al.* 2013). Beyond that, much less is known about the genetic causation of human NTDs, especially in cilia-related genes (Harris and Juriloff 2010).

To explore the potential role of cilia-related genes in NTD, we performed target gene next-generation sequencing (NGS) on candidate genes *BBS4*, *CEP290*, *DNAAF1*, *IFT172*, *INTU*, *MKS1*, and *IQCB1*. The protein encoded by the *DNAAF1* gene is required for the stability of the ciliary architecture and plays a role in cytoplasmic preassembly of distinct dynein-arm complexes, including outer dynein arms (ODAs) and inner dynein arms (IDAs) (Lee 2011), which are essential for motile cilia motility (Loges *et al.* 2009). Furthermore, DNAAF1 was expressed in some key tissues involved in neural system development, such as the neural tube, floor plate, embryonic node, and brain ependyma epithelial cells in zebrafish and mice (van Rooijen *et al.* 2008; Loges *et al.* 2009; Essner *et al.* 2005).

By screening a total of 373 Chinese NTD patients and 222 healthy controls, we detected eight disease-specific rare mutations in *DNAAF1*. In cell-based assays, two *DNAAF1* mutants were found to either downregulate DNAAF1 expression or disrupt the cytoplasmic preassembly of the dynein-arm complexes. In clinical samples, *DNAAF1* mutants affected expression level of key NTC-related genes. Altogether, we propose a hypothesis that DNAAF1 is probably implicated in the process of NTC in humans.

## Materials and Methods

### Human subjects

A total of 373 patients were enrolled at multiple local hospitals in Shanxi, Liaoning, Heilongjiang, Jiangsu, and Tianjin Provinces. We enrolled individuals with NTDs who were assessed by clinical geneticists and were categorized into at least one of the following diagnostic groups: anencephaly, spina bifida (aperta or cystica), craniorachischisis, spinal dysraphism, or encephalocele. A total of 222 ethnicity- and geography-matched controls were from nonmedically related terminations and were free of any NTD. All samples were collected with informed consent from the patients or their families, according to the requirements of the Ethics Committee at the Capital Institute of Pediatrics (Beijing, China).

### Target gene selection and NGS

We sequenced 281 NTD candidate genes, which were classified into three groups: (1) genes playing roles in NTC, (2) genes proven to be closely related to NTD in animal models, and (3) candidate genes suspected to contribute to the risk of NTD. The candidate genes were summarized based on their function, and the total number of genes were listed in each category (Supplemental Material, Table S1). The regions sequenced included all exons, 2 kb upstream of the promoter region, and conservative exon-intron boundaries.

Genomic DNA was isolated from the individual human samples and extracted with the Blood and Tissue DNA Kit (QIAGEN, Dusseldorf, Germany) according to the manufacturer’s instructions. The concentration and purity of DNA were determined by light absorbance at 260 and 280 nm. DNA was fragmented, enriched for both coding regions and highly conserved regions (the referenced genomic information is from GRCh37/hg19 in UCSC), and sequenced on an Illumina GA II instrument (Illumina, San Diego, CA) by Genesky. The samples were prepared by following the manufacturer’s standard procedure using a Truseq DNA Sample preparation kit (Illumina). With the aid of an Agilent Custom enrichment array (Probe Code: BI426526171), the library was constructed with an Agilent Custom SureSelect Enrichment Kit. Custom capture oligonucleotides were designed using the SureDesign website of Agilent technologies. The capture production was enriched with the following cycling conditions: 98° for 30 sec, 10 cycles of 98° for 10 sec, 60° for 30 sec, 72° for 30 sec, and 72° for 5 min. Twelve libraries were pooled, and bridge amplification was conducted on cBot (Illumina) following the manufacturer’s standard cluster generation protocols.

### Variant filtering and bioinformatics analysis

The sequencing reads were aligned to the hg19 iteration of the reference human genome using the Burrows–Wheeler Aligner v6.4 (Li and Durbin 2010). Single nucleotide variant (SNV) calling was performed using both Genome Analysis Toolkit (GATK) (McKenna *et al.* 2010) and Varscan programs(Koboldt *et al.* 2012), and further filtered using a recommended threshold value (mapping quality > 30, base quality > 15, nonreference allele number > 3, and nonreference allele frequencies > 0.2). A minimum 20-fold coverage per base was achieved on average for 95% of the target region. The called SNV data were then combined and annotated with the Annovar program (Wang *et al.* 2010). The functional effect of nonsynonymous SNVs was assessed by PolyPhen-2 (Polymorphism Phenotyping; http://genetics.bwh.harvard.edu/pph2/index.shtml) and SIFT (Ng and Henikoff 2003; Adzhubei *et al.* 2010). Nonsynonymous SNVs with SIFT scores of <0.05 or PolyPhen-2 scores of >0.85 were considered as significant of damaging. To sort potentially deleterious variants from benign polymorphisms, perl scripts were used to filter the SNVs against those of dbSNP135. Any SNV recorded in dbSNP135 and with a minor allele frequency of ≥1% in Chinese population from the 1000 Genome Database was considered as benign polymorphism and therefore removed from subsequent analysis. The genomic structure of human *DNAAF1* was determined using the NCBI GenBank (*DNAAF1*: NG_021174.1 and NM_178452.4). Novel rare variants of *DNAAF1* were then confirmed by repeating PCR reactions, followed by resequencing of both DNA strands. The primer sequences are available upon request.

### Plasmids

The human *DNAAF1* cDNA fused to the GFP tag at its C-terminus was synthesized by OriGene and subcloned into the expression vector pCMV6-AC. The disease-associated amino acid changes were generated by PCR-based mutagenesis using wild-type human *DNAAF1* cDNA as a template and referred to for convenience as ‘mutants’. Each mutant plasmid was also generated by OriGene. Integrity and the presence of wild-type and mutant plasmids in the resultant constructs were confirmed by direct sequencing.

### Cell culture and transient transfection

HEK293T cells were cultured in Dulbecco’s Modified Eagle’s medium (DMEM) supplemented with 10% fetal bovine serum (FBS), 1% antibiotic, and 1% glutamine (Gibco). Cells were seeded in a 10 cm^2^ cell culture dish, and cultured in a 37° incubator with 5% CO_2_. Transfection was performed using the Lipofectamine 2000 reagent (Invitrogen), according to the manufacturer’s instructions. MDCK cells were cultured and transfected using the same method as for HEK293T cells. NE-4C cells, purchased from the Stem Cell Bank, Chinese Academy of Science, were cultured on six-well plates coated with 15 µg/ml poly-L-lysine 2 hr before passage, and cultured in Eagle’s MEM (Gibco) supplemented with 10% FBS, 1% GlutaMAX (Invitrogen), and 1% nonessential amino acids (Invitrogen) at 37° temperature with 5% CO_2_.

### Western immunoblotting

Protein extraction was performed at 4°. Cells were harvested 48 hr post-transfection, washed in phosphate-buffered saline (PBS), and lysed in 1% (vol/vol) Nonidet P-40 buffer (150 μM NaCl, 10 μM Tris-HCl, pH 8.0; 1 mM DTT, 2 mM EDTA, 0.5 mM PMSF, 1% complete protease inhibitor cocktail, in PBS) for 10 min. Cell extracts were cleared by centrifugation at 12,000 rpm for 30 min. Total lysate samples were resolved by 10% SDS-PAGE and subjected to Western blot analysis with rabbit polyclonal anti-DNAAF1 antibody (sc-133762, Santa Cruz Biotechnology), followed by anti-rabbit horseradish peroxidase-conjugated antibodies (ZSBio), and detected with the West Pico ECL kit (Thermo Scientific).

### Immunofluorescence staining

Cells were cultured on cover-slips for 4 d in serum-free medium, transfected with corresponding plasmid, and continuously cultured for another 48 hr in serum-free medium, then washed in PBS, fixed in 4% paraformaldehyde for 15 min, and permeabilized in 0.1% Triton/PBS for 10 min in a 4° icebox. After incubation with 5% BSA for 1 hr at room temperature, primary antibodies were added: DNAAF1 (sc-133762; Santa Cruz Biotechnology), DNAI2 (H00064446-M01; Abnova Corporation), and DNALI1 (ab87075; Abcam). Secondary antibodies used were goat anti-rabbit IgG H&L (Alexa Fluor 594) (ab150080; Abcam) and donkey anti-mouse IgG H&L (Alexa Fluor 594) (ab150108; Abcam). Nuclei were stained with DAPI (P36935; Life Technologies). Immunofluorescence microscopy was carried out using a Leica TCS SP8 confocal microscope.

### RNA extraction and real-time RT-PCR

Total RNAs were extracted using the TRIzol Reagent (Invitrogen) and the quality was assessed with a nanodrop-1000 (Thermo Scientific) and Quantity One v4.6.3 system (Bio-Rad). RNA was then reverse-transcribed with the ProtoScript First Strand cDNA Synthesis Kit (E6300S; New England Biolabs). The levels of mRNA were determined using UltraSYBR Mixture (with ROX) (CW0956; CWBiotech). The levels of mRNA were normalized against the levels of *GAPDH*. The primers used were as follows: *DNAAF1*: forward 5′-TCGCACACCCAAGAGAAGAC3′, reverse 5′-GCGTATCATTCAATGCTGGGG-3′; *Lefty1*: forward 5′-ATACTGGGAGAGCTGGGGAT-3′, reverse 5′-GGGAACAATGGCAATTGGGG-3′; *Lefty2*: forward 5′-AGTGCTCTCGTCAGACCTTTG-3′, reverse 5′-TGGGGTCAGTGTGTCCTACT-3′; *Nodal*: forward 5′-GCCGACATCATTTGCCAGAC-3′, reverse 5′-CCCTCACAGCGATAGGCATT-3′; *Gli1*: forward 5′-CTGCGTGGTAGAGGGAACTC-3′, reverse 5′-GAAAGTCCTTCTGTTCCCATGC-3′; *Gli2*: forward 5′-ACTTTTGTCTCCTCGGGTCC-3′, reverse 5′-CTGCTGTCCTCCAAGAGACC-3′; *Ptch1*: forward 5′-GAAGCCACAGAAAACCCTGTC-3′, reverse 5′-GCCGCAAGCCTTCTCTAGG-3′; *Gapdh*: forward 5′-GTGGTGAAGCAGGCATCTGA-3′, reverse 5′-GCCATGTAGGCCATGAGGTC-3′. All PCR reactions were carried out using an Applied Biosystems 7500 fast PCR system.

### NanoString nCounter assay

Dissected brain tissue from the patient and control were homogenized in TRIzol Reagent (Invitrogen). Following the manufacturer’s instructions (Invitrogen), total RNA was extracted, and gene-specific probes were designed to the coding region of all 227 NTC-related genes by the manufacturer (NanoString Technologies). In addition, probes targeting clathrin heavy chain (*CLTC*), glyceraldehyde-3-phosphate dehydrogenase (*GAPDH*), glucuronidase-β (*GUSB*), hypoxanthine phosphoribosyltransferase 1 (*HPRT1*), and protein kinase cGMP-dependent type I (*PRKG1*) served as five internal reference genes. Scientific Support Services (UCL Genomics) performed the NanoString nCounter assay using RNA samples diluted to the appropriate concentration. Raw data in reporter code count (RCC) files were loaded into nSolver and used to perform quality assessment and normalization. Fold change of NTC-related genes expression in the patient was calculated compared to the control.

### Statistical analyses

Statistical analyses were performed using SPSS 20.0 program to calculate mean and SEM. Statistical significance of differences was evaluated by Student’s *t*-test. Values with a *P* < 0.05 (two-tailed analysis) were considered statistically significant.

### Data availability

The authors state that all data necessary for confirming the conclusions presented in the article are represented fully within the article.

## Results

### Rare variants identified in DNAAF1

With the aim of exploring novel genetic factors causing NTDs, we first screened a total of 373 NTD cases and 222 healthy controls using target gene NGS. This analysis identified 12 nonsynonymous amino acid changes within the cilia-related gene *DNAAF1*. Eight of them were found to be in nine NTD patients and were absent in 222 controls, the other four were identified only in controls. The mutation rate of *DNAAF1* was thus calculated to be 2.41% (nine of 373) in patients affected by NTDs. All variants were rare with a frequency of <0.005% (six of 120260) reported by the database of ExAC and ESP (Table 1). In East Asians, the mutation frequency found in *DNAAF1* was 7.63 × 10^−6^, based on the ExAC database. All nucleotide changes were found in the heterozygous form.

**Table 1:** Variants identified in *DNAAF1* through target gene NGS

No.	Nucleotide Change*^a^* (rs ID)	AA Change*^b^*	Property Change*^c^*	Conservation*^d^*	Domain*^e^*	REFQ*^f^* (1–8: Case 9-12: Control)	ExAC*^g^*	ESP*^h^*
1	c.371A > G	p.Asn124Ser	Polar (neutral) → polar (neutral)	Yes	Leucine-rich repeat	1/371	—	—
2	c.691A > C	p.Lys231Gln	Basic polar (positive) → polar (neutral)	Yes	Leucine-rich repeat	1/372	—	—
3	c.1022_1023delAA	p.Gln341Argfs*10	350 aa → premature termination codon	—	—	1/371	—	—
4	c.1135G > T	p.Val379Phe	Nonpolar (neutral) → nonpolar (neutral)	Yes	—	1/357	—	—
5	c.1786G > C	p.Glu596Gln	Acidic polar (negative) → polar (neutral)	Yes	—	1/372	—	—
6	c.1853G > A (rs372901697)	p.Arg618Gln	Basic polar (positive) → polar (neutral)	No	—	1/362	6/120260	1/12999
7	c.2054C > T	p.Ala685Val	Nonpolar (neutral) → nonpolar (neutral)	No	—	1/368	—	—
8	c.2060C > T	p.Ser687Phe	Polar (neutral) → nonpolar (neutral)	No	—	2/367	—	—
9	c.446C > T	p.Ala149Val	Nonpolar (neutral) → nonpolar (neutral)	Yes	Leucine-rich repeat	1/221	1/121406	—
10	c.721C > G	p.Leu241Val	Nonpolar (neutral) → nonpolar (neutral)	Yes	Leucine-rich repeat	1/221	—	—
11	c.1586C > G	p.Thr529Arg	Polar (neutral) → basic polar (positive)	No	—	1/221	—	—
12	c.2099C > A	p.Thr700Lys	Polar (neutral) → basic polar (positive)	No	—	1/219	1/121242	—

a *DNAAF1* GenBank RefSeq nos. NM_178452.4 and NG_021174.1. Nucleotide numbering reflects cDNA numbering with +1 corresponding to the A of the ATG translation initiation codon 1 in the reference sequence.

b The position of the mutations is given with reference to sequence accession NP_848547.4 for the protein.

c Amino acid residue property change.

d Amino acid residue evolutionary conservation.

e Location in protein secondary structure.

f Number of mutation carriers in case or control.

g The Exome Aggregation Consortium: http://exac.broadinstitute.org.

h NHLBI Exome Sequencing Project: http://evs.gs.washington.edu/EVS.

**Figure 1:**
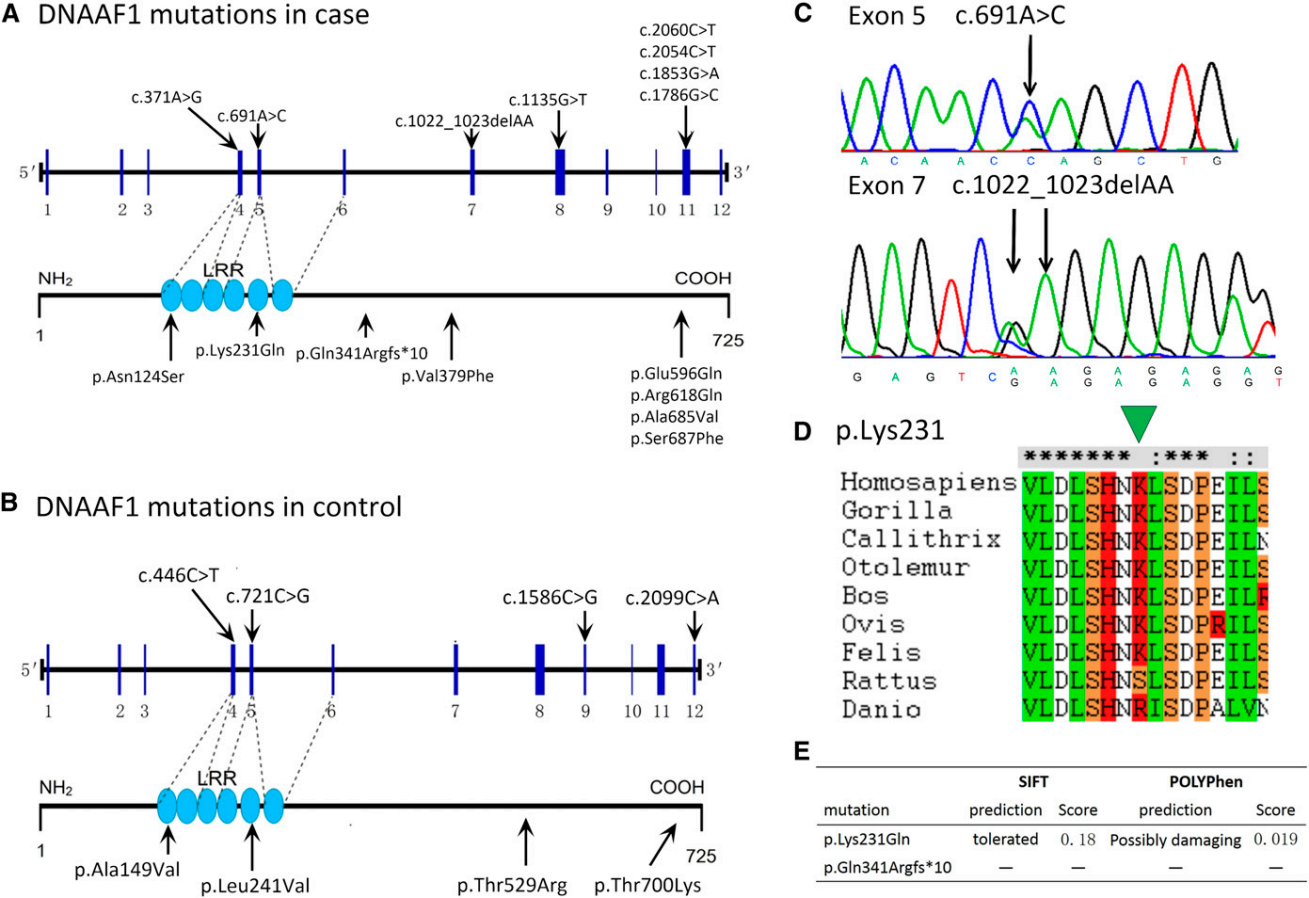
Rare nonsynonymous variants identified in *DNAAF1*. (A and B) Schematic representations of gene *DNAAF1* (RefSeq: NG_021174.1 and NM_178452.4) with the approximate locations of the rare nonsynonymous variants identified in the current study. (C) Mutations identified in two NTD patients were confirmed by repeating Sanger sequencing. (D) The evolutionary conservation of the p.231K. Clustal X protein sequence alignment of human DNAAF1 with orthologs from other species was displayed. The green arrowhead points to the residue K of 231 amino acid. (E) Functional prediction with SIFT and PolyPhen-2.

Eight nonsynonymous variants detected in NTD cases were: p.Asn124Ser (c.371A > G), p.Lys231Gln (c.691A > C), p.Gln341Argfs*10 (c.1022_1023delAA), p.Val379Phe (c.1135G > T), p.Glu596Gln (c.1786G > C), p.Arg618Gln (c.1853G > A), p.Ala685Val (c.2054C > T), and p.Ser687Phe (c.2060C > T) (Figure 1A). Among these mutations, one was a frameshift mutation c.1022_1023delAA in exon 7, derived from the deletion of two adenine nucleotides at positions 1022 and 1023 of the coding region that introduced a premature translation stop codon at position 350 (Figure 1, A and C). Seven were missense changes. Among them, p.Asn124Ser and p.Lys231Gln were located in the first and fifth leucine-rich repeat domain, respectively. Amino acid conservation analysis showed that four (p.Asn124Ser, p.Lys231Gln, p.Val379Phe, and p.Glu596Gln) mutations affected highly conserved amino acid residues, while others (p.Arg618Gln, p.Ala685Val, and p.Ser687Phe) involved less conserved amino acid residues (Table 1). p.Arg618Gln (dbSNP ID: rs372901697) was identified in healthy populations of East Asians, South Asians, and Europeans (http://exac.broadinstitute.org/variant/16-84209693-G-A). Four variants were found in controls: p.Ala149Val (c.446C > T), p.Leu241Val (c.721C > G), p.Thr529Arg (c.1586C > G), and p.Thr700Lys (c.2099C > A) (Table 1 and Figure 1B). Based on the bioinformatics data, including amino acid property change, conservation, and localization in key domains (leucine-rich repeat domain), we selected two mutations [p.Gln341Argfs*10 (c.1022_1023delAA) and p.Lys231Gln (c.691A > C)] for further functional study (Figure 1, D and E).

### Clinical features of NTD patients carrying DNAAF1 mutations

As shown in Table 2, all nine cases carrying *DNAAF1* mutations presented varying clinical features of NTDs. *DNAAF1* frameshift mutation p.Gln341Argfs*10 (c.1022_1023delAA) was identified in a male fetus (A1431) at 17 wk gestation, affected by anencephaly and meningomyelocele, two typical NTD phenotypes. The *DNAAF1* p.Asn124Ser (c.371A > G) substitution was found in a male fetus (SY657) with meningoencephalocele at 36 wk gestation. A 5-yr-old female patient (SY656) carrying the *DNAAF1* p.Ala685Val (c.2054C > T) variant also presented with meningoencephalocele. The *DNAAF1* p.Lys231Gln (c.691A > C) substitution was detected in a male fetus at 38 wk gestation (A1444) that presented with hydrocephalus, open lumbar sacral spina bifida, equinovarus, absence of skull, pulmonary lobe malformation, and horseshoe kidney. The *DNAAF1* p.Val379Phe (c.1135G > T) mutation was identified in a male fetus at 40 wk gestation (A2147) that presented with hydrocephalus, nonopen lumbar sacral spina bifida, equinovarus, pneumorrhagia, meconium aspiration syndrome, accessory spleen, and visceral congestion. The *DNAAF1* p.Glu596Gln (c.1786G > C) mutation was identified in a female fetus at 20 wk gestation (A2171) that presented with hydrocephalus, open thorac lumbar sacral spina bifida, equinovarus, visceral congestion, and atelectasis. Although the known *DNAAF1* missense variant p.Arg618Gln (c.1853G > A; dbSNP: rs372901697) was previously identified in the healthy population (0.0077%), in this study, a patient (SY2621) carrying the same variant, with unknown gestational age and gender, showed open lumbar sacral spina bifida. Two other female fetuses (A2097 and A1345) had the same mutation, *DNAAF1* p.Ser687Phe (c.2060C > T). Although both of them presented with spina bifida, there were phenotypic variations: A2097 had open cervical occipital thorac spina bifida, while A1345 had nonopen lumbar sacral spina bifida. Besides spina bifida and other systemic defects, A2097 was also affected by anencephaly, the most severe type of NTD, and A1345 was affected by hydrocephalus, which is commonly associated with spina bifida (Copp and Greene 2013), To summarize, six patients (A1444, A2147, A2171, SY2621, A209, and A1345) presented with varying degrees of spina bifida and four (A1444, A2147, A2171, and A1345) of the six were also affected by hydrocephalus (Table 2).

**Table 2:** Clinical features and genetic findings in patients with NTDs*^a^*

	Patient ID								
	SY657	A1444	A1431	A2147	A2171	SY2621	SY656	A2097	A1345
Mutations	p.Asn124Ser	p.Lys231Gln	p.Gln341Argfs*10	p.Val379Phe	p.Glu596Gln	p.Arg618Gln	p.Ala685Val	p.Ser687Phe	p.Ser687Phe
Gender	M	M	M	M	F	Unknown	F	F	F
Gestational age (wk)	36	38	17	40	20	Unknown	5Y*^b^*	16	20
Meningoencephalocele	+		+				+		
Hydrocephalus		+		+	+				+
Spina bifida		+*^c^*		+*^d^*	+*^e^*	+*^c^*		+*^f^*	+*^d^*
Anencephaly			+					+	
Equinovarus		+		+	+				
Absence of skull		+							
Pulmonary lobe malformation		+							
Horseshoe kidney		+							
Pneumorrhagia				+					
Meconium aspiration syndrome				+					
Accessory spleen				+					
Visceral congestion				+	+			+	
Atelectasis					+				
Pulmonary agenesis								+	
Postmortem autolysis								+	

a This table summarizes the clinical findings in the study participants.

b The patient is 5 yr old.

c Open lumbar sacral spina bifida.

d Nonopen lumbar sacral spina bifida.

e Open thorac lumbar sacral spina bifida.

f Open cervical occipital thorac spina bifida.

### DNAAF1 c.1022_1023delAA protein expression was deficient

**Figure 2:**
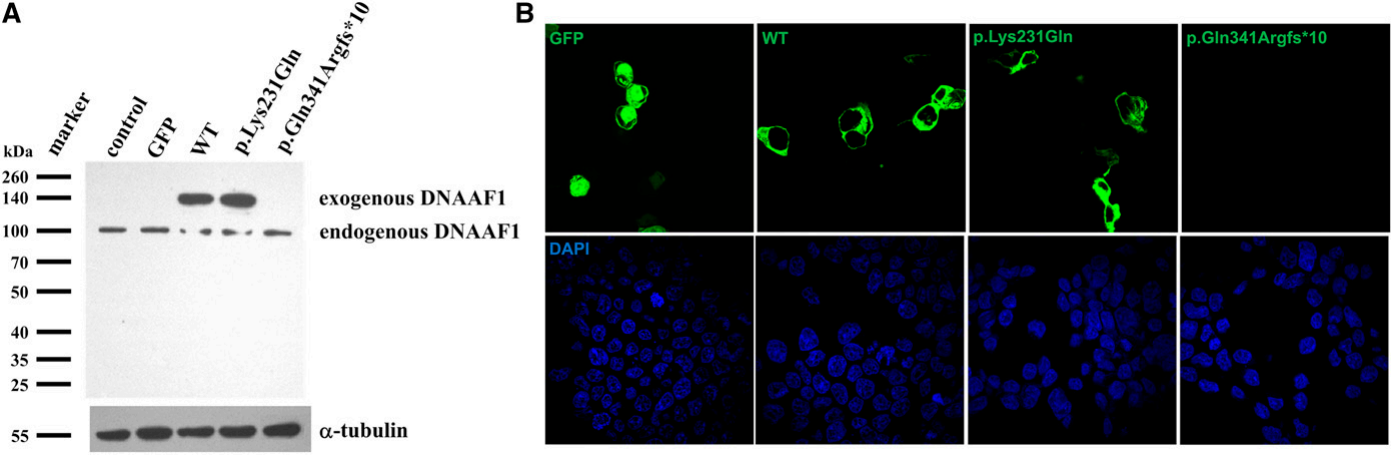
Protein expression and location of wild-type (WT) and mutant DNAAF1. (A) Expression of GFP-tagged DNAAF1 protein is shown by a Western blot analysis of whole cell lysate with anti-DNAAF1. α-tubulin was used as a loading control. Protein expression level of DNAAF1 c.691A > C was almost identical with the wild type, while DNAAF1 c.1022_1023delAA could hardly be detected. (B) Immunofluorescence staining showing protein location. Both c.691A > C mutant and wild-type GFP-tagged DNAAF1 protein localized in the cytoplasm, whereas no green immunofluorescence was detected in c.1022_1023delAA mutant transfected HEK293T cells. Transfected cells are marked by GFP (green).

Given that the c.1022_1023delAA frameshift mutation is predicted to cause premature termination of DNAAF1, we first tested whether intact DNAAF1 protein expression could be derived from a *DNAAF1* gene carrying such mutation. Wild-type or mutant DNAAF1 plasmids were transiently transfected into HEK293T cells and the protein expression level was examined using Western immunoblotting with antibodies specific to DNAAF1. The expression level of DNAAF1 c.1022_1023delAA was significantly diminished compared with that of the wild type, and that of DNAAF1 c.691A > C (p.Lys231Gln) was almost unchanged (Figure 2A). Fluorescence detection showed that both wild-type and missense mutant proteins were expressed in the cytoplasm, whereas no green fluorescence was detected in DNAAF1 c.1022_1023delAA plasmid-transfected HEK293T cells, indicating that no DNAAF1 protein was expressed in c.1022_1023delAA mutant cells (Figure 2B).

### DNAAF1 mutation leads to axonemal defects of the IDA (DNALI1) in MDCK cells

**Figure 3:**
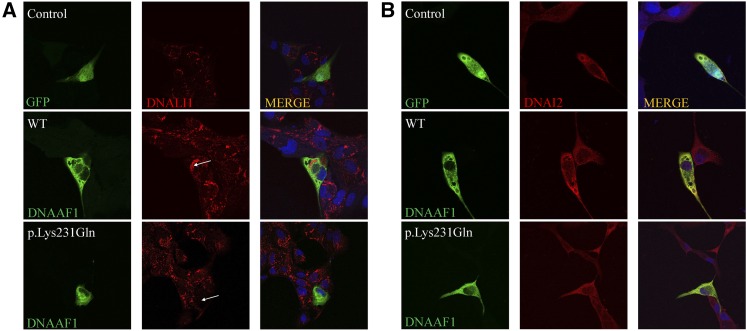
Axonemal defects of the IDA (DNALI1) in MDCK cells transfected with DNAAF1 missense mutant. High-resolution immunofluorescence microscopy of MDCK cells was performed with antibodies directed against the IDA component DNALI1 (A) and ODA chains DNAI2 (B). In control and GFP-tagged DNAAF1 wild-type (WT) transfected cells, DNALI1 and DNAI2 localize within the cytoplasm, whereas DNALI1 labeling is absent from the DNAAF1 p.Lys231Gln mutant transfected MDCK cells. The staining pattern of DNAI2 (B) is not obviously changed for the mutant. Nuclei were stained with DAPI (blue). GFP and GFP-tagged DNAAF1 protein are shown in green. DNALI1 and DNAI2 are shown in red.

To further assess the function of missense mutation in DNAAF1, we next analyzed MDCK cells cotransfected with various DNAAF1 expressing plasmids along with the corresponding plasmids for the expression of ODA protein DNAI2 (orthologs of *Chlamydomonas* dynein intermediate chain IC2) and the IDA component DNALI1 (ortholog of *Chlamydomonas* p28). Using high-resolution immunofluorescence microscopy, we observed complete absence of the IDA light chain DNALI1 from p.Lys231Gln mutant cotransfected cells, compared with control and wild type, demonstrating that the DNAAF1 p.Lys231Gln mutation disrupted cytoplasmic preassembly of the dynein-arm complexes (Figure 3A). However, no obvious alteration was detected for DNAI2 (Figure 3B).

### DNAAF1 mutants alter the expression of NTC-related genes and left–right patterning genes

**Figure 4:**
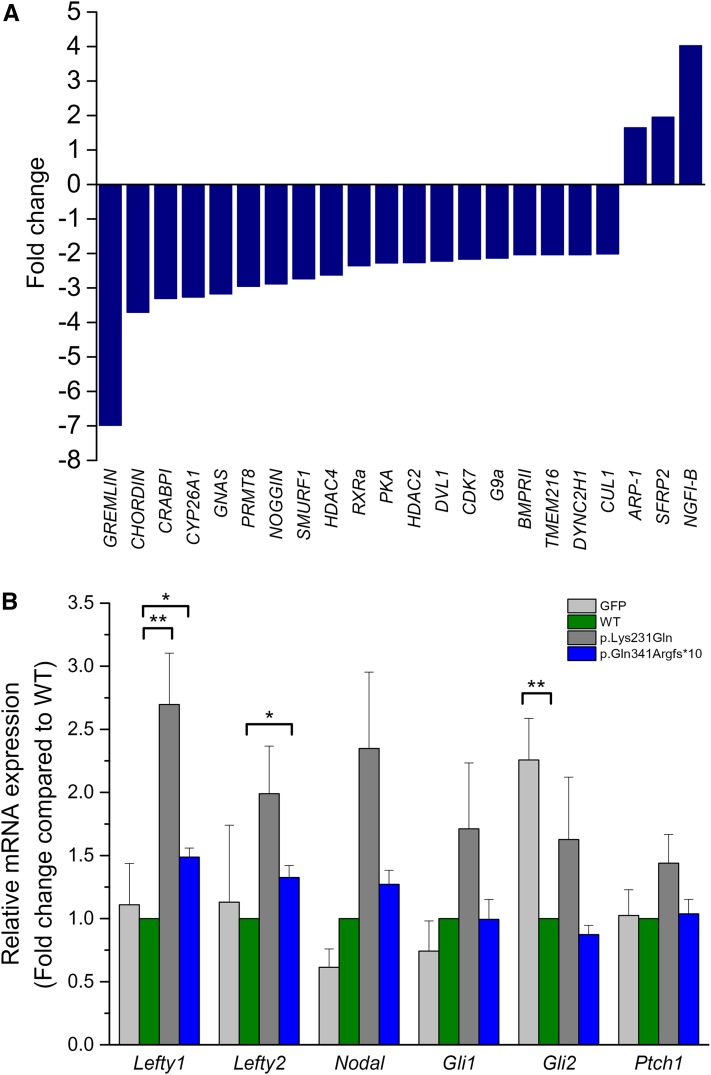
DNAAF1 mutants alter the expression of neural development related genes. (A) Changes of NTC-related gene expression in DNAAF1 mutant and control brain tissues, measured by NanoString, presented as bar charts on a fold change scale. Relative transcript data compared to control are shown for each distinct gene. (B) Left–right patterning gene expression was examined in DNAAF1 wild-type (WT) and mutant transfected NE-4C cells using the real-time PCR analysis. Data were normalized to *Gapdh* levels and expressed relative to the expression of wild type. Shown are mean ± SEM, *n* = 3. The asterisk indicates a statistically significant difference in comparison with wild type (* *P* < 0.05; ** *P* < 0.01).

Finally, in order to examine whether DNAAF1 contributes to neural system development, mRNA expression profiles of 227 NTC-related genes (Harris and Juriloff 2007, 2010) were evaluated in patient A1444 and control brain tissues by means of NanoString nCounter assay. Because patient A1431 with the *DNAAF1* p.Gln341Argfs*10 mutation exhibited anencephaly, we could not obtain the corresponding brain tissue. Compared to samples from healthy controls, three genes were upregulated and 19 downregulated, in samples from patient A1444 (Figure 4A).

Because DNAAF1 mutations cause the dynein arm defects of motile cilia (Loges *et al.* 2009), next we evaluated expression level of left–right patterning and SHH signaling-related genes in NE-4C neuro-ectodermal stem cells. Real-time PCR analysis revealed that level of the SHH target gene *Gli2* was decreased in NE-4C *DNAAF1* wild-type transfected cells compared with that of GFP mock cells (Figure 4B). Compared with the wild-type *DNAAF1* transfected cells, *Lefty1* was upregulated significantly in both p.Lys231Gln and p.Gln341Argfs*10 transfected cells, while *Lefty2* was significantly upregulated only in the p.Gln341Argfs*10 group (Figure 4B). Taken together, these results indicate that DNAAF1 plays a role in regulating gene expression and its mutation could lead to alteration of the NTC-related gene expressions.

## Discussion

Based on their structure and function, cilia can be generally divided into two subtypes: primary (monocilium or sensory cilia) and secondary (multicilium or motile cilia) cilia (Satir and Christensen 2007). Primary cilia have widespread distribution in many cell types. They are short, 9+0 arranged microtubule doublets that lack axonemal dynein (and hence are immotile) and play important roles in signal transduction (Satir and Christensen 2007). As mentioned above, cilia-related genes, such as *Arl13b* (Caspary *et al.* 2007; Higginbotham *et al.* 2012), *C2cd3* (Hoover *et al.* 2008), *Ift172* (Huangfu *et al.* 2003; Huangfu and Anderson 2005), and *Dync2h1* (Huangfu and Anderson 2005), were relevant to primary cilia or both types of cilia. Primary cilia are essential in patterning the neural tube (Murdoch and Copp 2010; Mariani and Caspary 2013; Goetz and Anderson 2010; Louvi and Grove 2011; Bay and Caspary 2012). In contrast, secondary cilia are much longer, 9+2 axonemal complexes with dynein arms beating cooperatively in wave-like patterns to power ciliary movement (Satir and Christensen 2007; Stubbs *et al.* 2008). In the neural system of vertebrates, secondary cilia, *i.e.*, motile monocilia or multiple motile cilia, are present in the embryonic node and the ependyma of the brain ventricle epithelial cell surfaces and generate fluid movement (Fliegauf *et al.* 2005; Satir and Christensen 2007). Ciliary motility is required for cerebrospinal fluid flow (Banizs *et al.* 2005) and provides essential developmental signals for embryonic left–right pattern determination (Tanaka *et al.* 2005). Previous studies showed that mutations in the motile cilia gene *DNAAF1* cause primary ciliary dyskinesia (Loges *et al.* 2009) and seminoma (Basten *et al.* 2013). This is, to our knowledge, the first report that describes mutations in *DNAAF1* identified among human patients with NTDs. In particular, consistent with previous conclusions that hydrocephalus is a very common association with spina bifida (Copp and Greene 2013), four out of the nine patients affected by spina bifida also displayed hydrocephalus, suggesting that variants in DNAAF1 might be associated with NTDs.

In a cohort of 373 NTD patients, we identified eight nonsynonymous mutations in *DNAAF1* affecting nine NTD patients, which were absent in the 222 controls. All nine cases with different *DNAAF1* mutations presented varying clinical features of NTDs, *e.g.*, anencephaly, meningomyelocele, varying degrees of spina bifida, and hydrocephalus. Based on data from bioinformatics analysis, we predicted that two of the eight mutations (p.Gln341Argfs*10 and p.Lys231Gln) might have a disruptive role in protein function and therefore were selected for further functional analysis.

First, we showed that the *DNAAF1* p.Gln341Argfs*10 mutant produced no detectable level of DNAAF1 protein in transfected HEK293T cells, as expected, while the p.Lys231Gln mutant had a similar level of expression as the wild type. However, immunostaining indicated that the missense mutant may disrupt cytoplasmic preassembly of the dynein-arm complexes with the absence of the IDA light chain DNALI1. These findings demonstrate that the novel mutations of *DNAAF1* identified in this study may cause the loss of function and dysmotility of the motile cilia. Next, we conducted experiments to find the possible relationship between *DNAAF1* mutation and NTDs. *DNAAF1* is a key structure gene of the motile cilia, but very little information had been reported on motile cilia and neural tube development, except for their occasional roles in the formation of the hydrocephalus (Banizs *et al.* 2005). Previous research on motile cilia-related gene *Foxj1* indicated that *Foxj1* could affect NTC by decreasing *Ptch1* and *Gli1* expression, the transcriptional mediators of the SHH signaling. This function of *Foxj1* relied on the architecture and morphology of motile cilia (Cruz *et al.* 2010). It suggests that motile cilia in the neural tube, nodal, floor plate, and ependyma might directly regulate gene expression by itself with the transcriptional activity, or through fluid flow induced by the cilia movement, and that change in the gene expression level would result in NTDs. Thus, we hypothesize that the defective function of motile cilia caused by *DNAAF1* mutation may lead to alteration in gene expression. Therefore, we evaluated mRNA expression profiles of 227 NTC-related genes in the brain tissues of patient A1444 and a control by means of NanoString nCounter assay. Our results demonstrated that there were three genes upregulated and 19 genes downregulated in patient A1444 compared to control. In addition, by transfecting the *DNAAF1* plasmids into NE-4C neural stem cells, we identified an altered expression of the left–right patterning genes *Lefty1* and *Lefty2* in the mutant groups. It should be noted that the data from NE-4C cells were not consistent with results in the brain, probably because the samples used were at different stages of neural development. NE-4C cells were cloned from the anterior brain vesicles of 9-d-old, p53^−/−^ mouse embryos (Schlett and Madarasz 1997), at the early stage of neural system development, whereas brain tissues we used were from 38 wk gestation, the last stage of neural system development. In addition, brain tissues *in vivo* were more complicated than cell cultures *in vitro*. These data demonstrated that DNAAF1 could affect NTC-related gene expression, which may finally influence the development of neural tubes. Further study is needed to identify the underlying mechanism through which this regulation is achieved.

In summary, our findings here demonstrate that mutations in the motile cilia gene *DNAAF1* could contribute to the pathogenesis of NTDs. The relevance of DNAAF1 to neural tube formation is demonstrated based on the following evidence: (1) DNAAF1 is expressed in the neural tube, floor plate, embryonic node, and brain ependyma epithelial cells during embryonic development (van Rooijen *et al.* 2008; Loges *et al.* 2009; Essner *et al.* 2005). (2) DNAAF1 plays a role in cytoplasmic preassembly of the dynein arms, and disruption of the ependymal cilia dynein arm assembly resulted in loss of cerebrospinal fluid flow and subsequent development of hydrocephalus (Mitchison *et al.* 2012; Kramer-Zucker *et al.* 2005). Defects in node cilia dynein arms may affect the nodal flow and result in randomization of left–right body asymmetry in zebrafish and human patients (Loges *et al.* 2009; Mitchison *et al.* 2012; Kosaki *et al.* 2004); (3) patients with mutation in *DNAAF1* in our study were affected by NTDs; (4) extremely rare mutations were identified in 2.41% (nine of 373) of Chinese NTD patients, and the two dissected ones clearly introduce loss-of-function; and (5) DNAAF1 mutants alter the expression of NTC-related genes and left–right patterning genes. Taken together, our findings refine DNAAF1 as a potential risk factor in human NTDs. Further studies are warranted to further elucidate the underlying mechanisms, and define to which extent this (and other) motile cilia gene may contribute to human NTD susceptibility.

## Supplementary Material

Supplemental Material
