# Supplementary material for: Mutations in the Motile Cilia Gene DNAAF1 Are Associated with Neural Tube Defects in Humans
Source: G3 (Bethesda). 2016 Aug 18;6(10):3307–16. doi: 10.1534/g3.116.033696 (PMC5068950; doi:10.1534/g3.116.033696)
Supplement: Supplemental Material [file supp_6_10_3307__index.html]

Mutations in the Motile Cilia Gene DNAAF1 are Associated with Neural Tube Defects in Humans — Mutations in the Motile Cilia Gene DNAAF1 are Associated with Neural Tube Defects in Humans — Mutations in the Motile Cilia Gene DNAAF1 Are Associated with Neural Tube Defects in Humans — Supplemental Material 

# Mutations in the Motile Cilia Gene *DNAAF1* Are Associated with Neural Tube Defects in Humans

## Supplemental Material for Miao, *et al*, 2016

**Files in this Data Supplement:**

- Table S1 - A summary for the candidate genes sequenced in the current study. (.pdf, 8 KB)
