# Supplementary material for: Mutations in the Motile Cilia Gene DNAAF1 Are Associated with Neural Tube Defects in Humans
Source: G3 (Bethesda). 2016 Aug 18;6(10):3307–16. doi: 10.1534/g3.116.033696 (PMC5068950; doi:10.1534/g3.116.033696)
Supplement: Supplemental Material [file supp_g3.116.033696_TableS1.pdf]

Table S1. A summary for the candidate genes sequenced in the current study

Pathway	Gene
Planar cell polarity signaling	20 genes, including CELSR1, DVL1, FUZ etc.
Cytoskeleton	21 genes, including ABI1, SHROOM3, VCL etc.
Canonical Wnt signaling and Wnt-$Ca2^{+}$ signaling	11 genes, including FZD4, LRP6, CTNNB1 etc.
Homeobox related	10 genes, including HOXA1, PAX3, SIX3 etc.
Cilia	7 genes, including BBS4, CEP290, DNAAF1, IFT172, INTU, MKS1, IQCB1
Cell cycle	3 genes, including OFD1, STIL, GMNN
Neural development	16 genes, including ALX1, ZIC2, TRPM6 etc.
TGF-beta signaling	8 genes, including BMP4, NOG, ZEB2 etc.
One carbon metabolism	32 genes, including FOLR1,MTHFD1, MTRR etc.
Notch signaling	9 genes, including TWIST1, MIB2,NUMB etc.
Hedgehog signaling	8 genes, including PTCH1, RAB23, SHH etc.
Apoptosis	14 genes, including APAF1, CASP3, BCL10 etc.
MAPK signaling	14 genes, including MAPK9, NF1,PRKACA etc.
DNA repair and damage	12 genes, including BRCA1, HIPK1, TERC etc.
Chromatin modifier	24 genes, including DNMT3B, EP300,CITED2 etc.
ECM and cell adhesion	17 genes, including ITGA6, FREM2, EPHA7 etc.
Glucose metabolism	15 genes, including POMT1, FOXO1, TSC1 etc.
Retinoid metabolism	6 genes, including ALDH1A2, CYP26A1, RARA etc.
Inositol metabolism	4 genes, including INPP5E, TULP3, PIP5K1C etc.
Protein processing	8 genes, including NUP50, HECTD1, TCOF1 etc.
Others	22 genes, including SP8, LUZP1,CSK etc.
